# Oral Rehabilitation from Oral and Dental Diseases

**DOI:** 10.3390/healthcare10102065

**Published:** 2022-10-18

**Authors:** Yoichiro Ogino

**Affiliations:** Section of Fixed Prosthodontics, Division of Oral Rehabilitation, Faculty of Dental Science, Kyushu University, 3-1-1 Maidashi, Higashi-ku, Fukuoka 812-8582, Japan; ogino@dent.kyushu-u.ac.jp; Tel.: +81-92-642-6371

Oral conditions, such as the number of teeth and oral hygiene, are related to oral functions and oral health-related quality of life (QoL) [1,2]. Furthermore, oral conditions, especially oral functions, have been known to play a crucial role in systemic health (physical and mental functions) [3]. This means that oral conditions are generally associated with not only oral health-related QoL, but also general QoL, defined as “an individual’s perception of their position in life in the context of the culture and value systems in which they live and in relation to their goals, expectations, standards and concerns” by World Health Organization [4]. Oral conditions are generally disrupted by oral and dental diseases, including caries, periodontal disease, tooth loss, oral mucosal lesions, and oral cancers. Notably, oral frailty and oral sarcopenia, regarded as the age-related poor conditions in oral health, are related to oral hypofunction. These conditions may result in dysphagia and malnutrition, followed by physical and cognitive impairment [5,6,7,8,9]. These imply that oral conditions and oral functions are key factors to maintain better QoL.

Oral rehabilitation to improve oral conditions includes dental treatments, such as operative dentistry, periodontal therapy, and prosthodontic treatment. The previous reports analyzed the outcomes of their interventions. These outcomes generally depended on the interventions, which included the clinical evaluation of some parameters, survival and success rates, and the prognosis of intervention. In addition, the exercises of perioral muscles, including the tongue, has been of increasing interest [10,11,12,13,14,15]. Based on the significant effects of oral conditions and oral functions on systemic conditions, it must be very important to show the effects of the novel oral rehabilitations on dental and oral diseases, oral functions, and systemic conditions from multiple points of view, and to review the etiology, treatment procedures, and the clinical impacts of oral rehabilitation on specific oral diseases and conditions. 

The aim of this special issue entitled “Oral Rehabilitation from Oral and Dental Diseases” is to introduce the current research on oral rehabilitation and reconsider previous reports in this field.

## Data Availability

Not applicable.
